# Health Information Technology and Accountable Care Organizations: A Systematic Review and Future Directions

**DOI:** 10.5334/egems.261

**Published:** 2019-07-08

**Authors:** Casey P. Balio, Nate C. Apathy, Robin L. Danek

**Affiliations:** 1 Indiana University Richard M. Fairbanks School of Public Health, US; 2 Regenstrief Institute, US; 3 Indiana University School of Medicine, Terre Haute, Rural Medical Education Program, US

**Keywords:** health information technology, electronic health records, health information exchange, accountable care organization, Patient Protection and Affordable Care Act, Health Information Technology for Economic, Clinical Health Act

## Abstract

**Background:**

Since the inception of Accountable Care Organizations (ACOs), many have acknowledged the potential synergy between ACOs and health information technology (IT) in meeting quality and cost goals.

**Objective:**

We conducted a systematic review of the literature in order to describe what research has been conducted at the intersection of health IT and ACOs and identify directions for future research.

**Methods:**

We identified empirical studies discussing the use of health IT via PubMed search with subsequent snowball reference review. The type of health IT, how health IT was included in the study, use of theory, population, and findings were extracted from each study.

**Results:**

Our search resulted in 32 studies describing the intersection of health IT and ACOs, mainly in the form of electronic health records and health information exchange. Studies were divided into three streams by purpose; those that considered health IT as a factor for ACO participation, health IT use by current ACOs, and ACO performance as a function of health IT capabilities. Although most studies found a positive association between health IT and ACO participation, studies that address the performance of ACOs in terms of their health IT capabilities show more mixed results.

**Conclusions:**

In order to better understand this emerging relationship between health IT and ACO performance, we propose future research should consider more quasi-experimental studies, the use of theory, and merging health, quality, cost, and health IT use data across ACO member organizations.

## Introduction and Background

Accountable care organizations (ACOs), one of the delivery system reforms set in motion by the Patient Protection and Affordable Care Act (PPACA), have continued to grow in number [1]. Currently more than 900 ACOs have formed and cover 32.4 million Americans [1] and in 2015, 20 percent of hospitals were participating in an ACO [2]. ACOs establish contractual agreements for coverage of a population across a variety of health care delivery organizations. Those organizations agree on financial incentives to coordinate high quality care, produce better population health outcomes, and reduce costs [3,4]. Seeking evidence on the success of these new care models, health services researchers have focused on ACOs to better understand if and how these organizations are achieving these goals.

Early conceptions of ACOs in the late 2000s cited the organizational capabilities required for success [5,6,7], and subsequent literature has highlighted the general belief that this success will depend in part on effective health IT [8,9]. This work emphasized two key mechanisms for success: first, exchange of electronic health data (EHD) among organizations to coordinate care and second, performance tracking within an ACO. To these ends, ACOs could use EHD to measure physician performance, inform quality improvement initiatives, identify population health needs, and facilitate clinical information exchange [10,11]. These capabilities largely depend on the implementation and continued development of health information technology (IT) efforts among the member organizations, especially adoption of electronic health records (EHR) and use of health information exchange (HIE). This relationship combined with the policy-driven rise in EHR adoption [12], slow rise in HIE among hospitals [13,14], and ACO formation [1] creates a favorable environment for studying the intersection of health IT and ACOs.

Subsequent research has explored the relationships between health IT adoption and ACO participation, but unifying themes have been difficult to assess. Studies vary in the organizational unit of analysis: some study physician groups [8,15,16], others focus on hospitals [17,18,19], and many study the ACO as a single organization [20,21,22,23,24,25,26]. Beyond the organizational unit, health IT is measured differently, ranging from EHR adoption [15,22,27] to implementation levels [17,24] and more specific measures of EHR capabilities [18,20,25,28]. Finally, studies differ in their treatment of health IT as an input [15,17,18,27] or an outcome [20,24,25], and have mixed findings within those subgroups. Given the heterogeneity in studies within this literature, a careful orientation and synthesis of the existing literature is necessary. By doing this, we aim to establish the needs for the next chapter of research questions exploring the specific mechanisms through which health IT can contribute to ACO performance.

The primary purpose of this paper is to review and synthesize the ongoing work regarding ACOs and health IT. We also propose future priorities for this stream of literature regarding study design, data sources, and use of theory. We conducted a systematic literature review in order to assess the current evidence and ongoing research. Our findings can assist leaders of ACOs in making evidence-based decisions regarding health IT. Additionally, this information will be useful to researchers seeking to contribute to this body of evidence. Finally, our suggestions for future research will be of interest to policymakers evaluating ACO performance and organizations involved in the collection and dissemination of research data sets.

## Methods

We searched PubMed and MEDLINE for all studies examining both ACOs and health IT. We used broad keywords to ensure the largest pool of studies in our initial search. We defined health IT as any use of technology within the context of health care to provide care, coordinate care, improve internal processes, or report to regulatory agencies. Any use of health IT that fit into these use cases qualified for inclusion. We required that studies identified ACOs formally. Studies investigating organizations with more general approaches to coordinated care or system integration that were not formally identified as ACOs did not qualify for review. Similarly, we did not include studies citing relevance of their findings for ACOs, as these did not directly investigate organizations formally established as ACOs. Finally, studies discussing ACOs as delivery reform but not explicitly measuring or analyzing ACO participation were excluded from the review.

### Data Sources and Searches

Searches were conducted in PubMed and MEDLINE. To improve the comprehensiveness of our search, we conducted reference snowballing on all papers in the full-text review stage until no new studies appeared in references. We also employed a hand-search methodology to augment the search and reference snowballing. Two investigators (CB & NA) evaluated each study for inclusion, and disagreements were resolved via consensus. Each investigator extracted study information using a standard form, and data collection was reviewed by both for completeness and accuracy. We classified studies into three streams, and further subdivided individual outcomes into different streams if needed.

The relevant stream for each study or outcome was determined based on the relationship between the variables assessing health IT and ACO status. The first stream included studies of health IT as a determinant of ACO participation, with ACO participation or formation as the outcome variable. The second stream included studies in which ACO participation was either a control variable or independent variable of interest, with health IT as the outcome variable. Finally, the third stream included studies that examined health IT as a determinant of ACO performance measures (as distinct from participation and formation).

### Data Synthesis

The studies we identified could not be meta-analyzed due to the heterogeneity in measurement and study design. In addition, within-stream heterogeneity of studies prevented us from meta-analyzing studies within each of the three streams. In light of this, we took a qualitative analytic approach.

## Results

**Figure 1:**
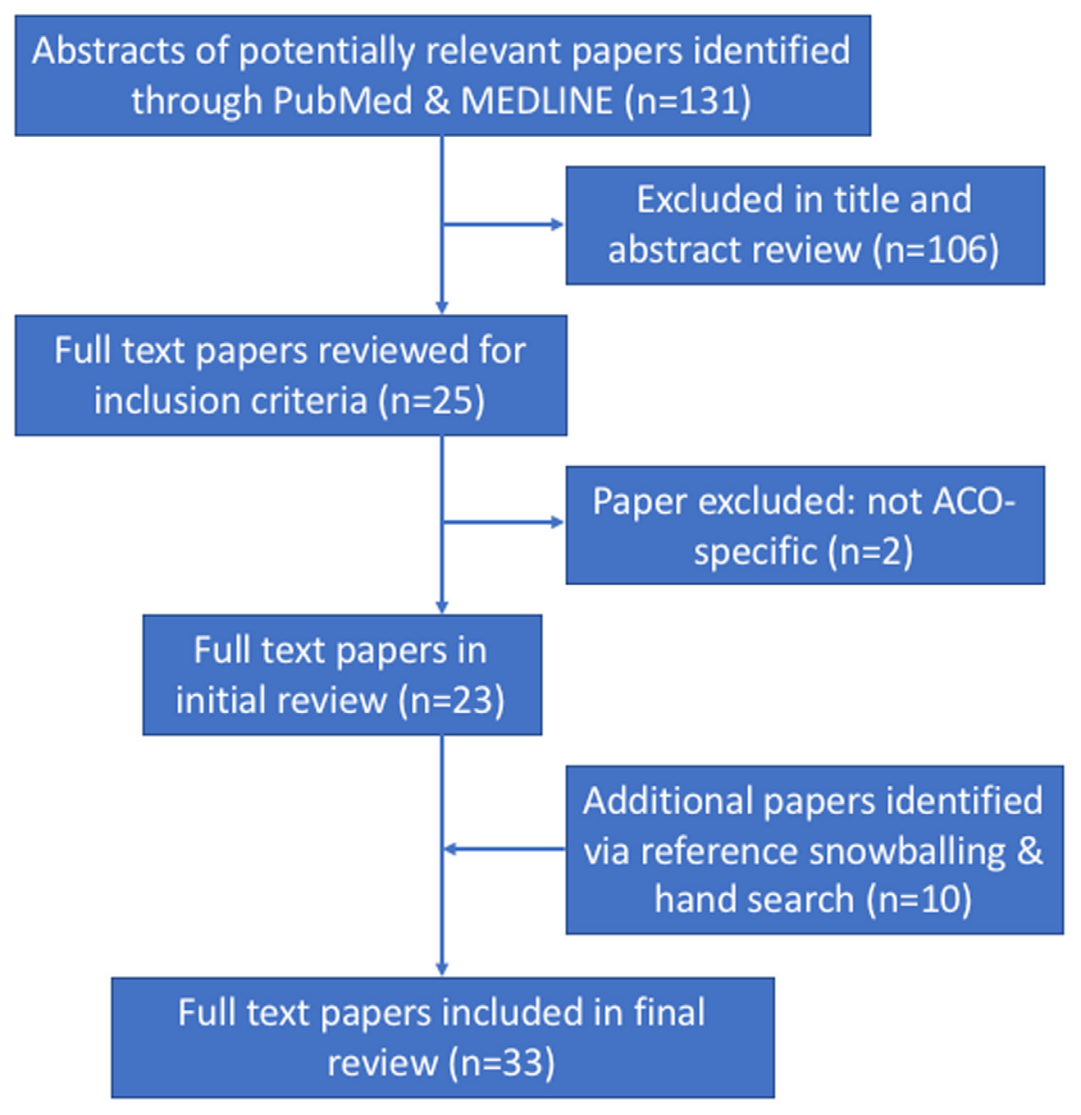
PRISMA Flow Diagram.

From PubMed, we identified 131 papers meeting our search criteria (Figure 1). After title and abstract review, there were 25 studies exploring ACOs and health IT. We performed full-text review on each of these 25 and eliminated three additional studies due to a lack of focus on ACOs as a formal organizational type of interest. From the 22 remaining studies, we identified 10 additional studies via reference snowballing and additional hand search, for a final count of 32 studies included in the review.

In order to synthesize this diverse body of literature, studies were grouped into streams based on one of three purposes: health IT as a determinant of ACO participation, health IT as an outcome, and how health IT relates to ACO performance (Table 1). We explore the study types, data, and findings for each of these streams below.

**Table 1:** Summary of Streams.

	Main Findings	References

**Stream 1:** Health IT as a Determinant of ACO Participation	With some exceptions, health IT is generally positively associated with ACO participation and qualitative studies often report health IT as a reason for participation or lack of capabilities as a barrier for participation.	Ortiz, 2013^+^ Wan, 2014^+^ Colla, 2015* Yeager, 2015^+^ Heisey-Grove & Patel, 2017^+^ Walker, 2016^+^ Chukmaitov, 2017^+^ Cross, 2017^+^ Lewis, 2017 (*Soc Sci Med*)*
**Stream 2:** Health IT Use by ACOs	Many ACOs report having an EHR or HIE capabilities but specific advanced capabilities are generally reported by fewer than 50% of ACOs.	Colla, 2014* DuBois, 2014* Shortell, 2015* Colla, 2016* Heisey-Grove & Patel, 2017^+^ Peiris, 2016* Wu, 2016 (*J Health Organ Manag*)* Ali, 2017^+^ Bazzoli, 2017* Heisey-Grove & King, 2017^+^ Lewis, 2017 (*Med Care Research & Rev*)* Markovitz, 2017* Pimperl, 2017^+^ Wilks, 2017*
**Stream 3:** ACO Performance and Health IT Use	Qualitative: ACO and member organization leaders generally agree that health IT is important for their organizational goals and cost and quality incentives. Quantitative: Some evidence of improved care processes, reduced costs and 30-day mortality while other studies find no relationship to cost or quality outcomes.	Larson, 2012* Albright, 2016* D’Aunno, 2016* Schoenhaus, 2016* Stock, 2016* Wu, 2016 Bagwell, 2017* King, 2016^+^ Huber, 2017^+^ Kim, 2017* Wilks, 2017* Wu, 2017*

*Note*: For summaries of each study, see Appendix Table 2. * Indicates study sample for the extracted finding reflected ACOs or organizations or providers only within ACOs. ^+^ Indicates study sample was of organizations or providers and further considered differences between those participating in an ACO or not.

### Stream 1: Health IT as a determinant of ACO Participation or Formation

Several studies consider health IT as a determinant of ACO participation. Within this stream, studies explore perceptions of ambulatory health care managers [27] and compare health IT use among physicians [15], rural health clinics [19], long-term care facilities [29], outpatient pharmacies [26], and hospitals [17,18,30] participating in ACOs. Although there are exceptions [18,26,27,30], studies across this stream have generally shown that greater health IT capacities (specifically using EHR and HIE) are associated with greater likelihood of participating in an ACO [15,17,18,29,30]. Nuanced variations in findings within individual studies and across this stream illustrate that the relationship between various forms of health IT and ACO participation is complex. For example, the role of health IT as a determinant of partnering to form the ACO [31] differs between Pioneer ACOs and the subsequent Medicare Shared Savings Program (MSSP) ACOs [30]. These differences highlight how study design, populations studied, and measures of technology use can impact our understanding of this relationship.

### Stream 2: Health IT as an Outcome

Similar to stream 1 which suggested hospitals and physicians using health IT are more likely to participate in ACOs, findings from stream 2 evaluate the inverse of this relationship. Findings from studies in stream 2 suggest that hospitals and physicians participating in an ACO are more likely to perform a variety of processes electronically and report more advanced health IT use. For example, ACO-affiliated physicians are more likely to perform several population management, patient engagement, and quality improvement activities electronically [15] and are more likely to integrate performance management systems into the EHR [32] than those not affiliated with an ACO. Two qualitative studies identified advanced health IT capabilities as being a reason for including a hospital in the ACO [2] and the use of health IT as a strategy for improving care transformation [33]. As strategies for reducing spending, readmissions, and redundant testing, a survey of emergency departments participating in ACOs found that 31 percent established telemedicine processes and 65 percent invested in their health IT infrastructure [34]. One of the few studies explicitly addressing health IT use among ACOs utilized mixed methods to provide early descriptions of health IT use [25]. These early findings suggest a wide range of health IT capabilities, from less than 10 percent of ACOs integrating outside data to 53 percent reporting EHR-based drug interaction checking [25]. When considering health IT use with regard to patient engagement efforts, over half of ACOs use telehealth and are able to send notifications to patients electronically, just under half (48 percent) provide patients with access to medical records, and an additional 24 percent provide patients with access to medical records and clinical notes [24]. However, aside from a few exceptions such as drug-drug/drug-allergy interaction checks, telehealth, and electronic notification sending, each of these studies found that fewer than 50 percent of ACOs reported participating in a variety of health IT activities [20,22,24,25,35,36].

Health IT capabilities may also differ depending on the organizational structure of the ACO, whether it is hospital or physician-led [20,25], and whether it is commercial [22] or not. Within Medicare ACOs, both Pioneer and MSSP ACOs generally had high basic and advanced health IT capabilities although some ACOs of each type were classified by their low health IT capabilities [37].

In addition to ACO participation being a determinant of health IT use, past experiences with various policies may also influence the relationship. A study of physician practices considered ACOs’ past experience with financial incentives and public reporting requirements as indicators of the organization’s preparedness for participation in value-based reform efforts such as ACOs [38]. Practices participating in ACOs with prior exposure to public reporting requirements were more likely to be prepared to implement Meaningful Use (MU, a policy incentivizing the use of health IT). Additionally, practices in ACOs with prior exposure to financial incentives and those with experience with public reporting requirements were more prepared to use quality and cost data from their organization. One study specifically identified providers’ participation in a Pioneer ACO as significantly associated with MU registration and attestation for both Medicaid and Medicare providers, although these providers were significantly less likely to receive funding for adoption, implementation, or upgrades [39]. This could be an indication of advanced health IT use prior to MU, making these funds less important for these early-adopter providers.

While stream 1 and stream 2 may appear to be in opposition of each other, it should be noted that studies are often cross-sectional in nature and therefore a causal relationship in either direction cannot be determined. Findings from streams 1 and 2 may reflect bidirectional causality or a confounded relationship between health IT and ACOs due to certain organizational characteristics, environmental factors, or something else which causes practices to participate in *both* health IT and ACOs.

### Stream 3: ACO Performance Indicators as a Function of Health IT

Some of the most recent studies have evaluated the association between clinical, cost or quality measures and ACO health IT capabilities. Studies of provider groups have found mixed results. One study identified no relationship between EHR functionality and care management activities [40], but another found that using Certified EHRs and participating in either an ACO or patient-centered medical home (PCMH) was associated with the highest odds of performing population management, care coordination, quality measurement, and communication compared to physicians using a Certified EHR but not participating in an ACO or PCMH [8]. Six qualitative ACO-level studies found that ACO leaders emphasized the importance of health IT capabilities in achieving their quality, population health, or financial goals [16,21,25,35,41,42]. Additionally, one study found that ACOs performing highly on quality and financial metrics reported extensive and sophisticated use of EHRs in their organizations as a factor for success [43].

Four quantitative studies have examined specific health IT capabilities and ACO performance measures. Positive associations were found between increased EHR capabilities and disease prevention [23]; information exchange and care management processes [28]; and integration of a new medication refill system and time and cost savings [44]. There was no evidence of significant relationships between either information capture or provision with care management processes [28]. While many ACO leaders site health IT as being integral for their success, empirical evidence supporting that claim at this point is mixed.

### Other Findings: Data Sources, Funding Mechanisms, and Theory

In addition to whether health IT was included as an input or output in the analysis, studies varied in terms of data sources, sample, funding mechanisms, and the use of theory. Frequently used data sources include the National Survey of ACOs (NSACO), annual American Hospital Association (AHA) survey data, Centers for Medicare & Medicaid Services (CMS) MSSP ACO performance and public use files, the National Study of Physician Organization Survey, and interviews with ACO leaders. These sources provide data from a variety of levels within an or outside of an ACO including the physician, physician group, hospital, and ACO level, resulting in a different unit of analysis. The sample of the study, as reflected in Table 1, may include any of these units of analysis described either within an ACO only or comparisons between those for ACOs and non-ACOs. The most common funding sources included the Robert Wood Johnson Foundation, Commonwealth Fund, various divisions of NIH, and AHRQ. Several institutional funding mechanisms were also present. Lastly, of the 33 studies reviewed, 14 used either a traditional organizational theory [17,24,27,28,31], early ACO frameworks or findings [20,21,22,30,37], or other policy or health frameworks [25,38,43] as a theoretical or conceptual framework. While there are some examples of multiple studies using the same theory or framework, there is generally a lack of repetition of frameworks used in this field, further contributing to the heterogeneity of work in this area.

## Discussion and Future Directions

Overall, our findings suggest that health IT capabilities are generally associated with increased formation of and participation in ACOs. Additionally, ACOs have greater health IT capabilities on average than non-ACO organizations. This reflects the findings from a national survey of providers from 2015 in which 92 percent of physicians in an ACO report using a certified EHR. Rates are similar among physicians participating in other delivery reform efforts, and overall while 90 percent of physicians participating in any delivery reform effort report using a certified EHR, only 68 percent of physicians not participating in a delivery reform effort report using a certified EHR [45]. Despite the generally consistent positive relationship between health IT and ACO participation (stream 1), evidence of the relationship between health IT and ACO performance (stream 3) is less conclusive. These mixed findings may be due to the still early experience with ACOs, difficulty linking necessary data sources to explore this relationship, or the implementation of ACO and health IT efforts nearly simultaneously, increasing the difficulty of more rigorous studies to assess causality. Studies also differ in which organizational level they assess (i.e. physician group, hospital, or whole ACO), what types of health IT they consider (i.e. EHR, HIE, pharmacy services, patient engagement), and the granularity of their measurement of health IT use. This heterogeneity is natural for such a relatively new research area, but makes understanding the evidence of this complex relationship difficult. Our findings suggest several imperatives for moving forward in this body of literature. Focusing on quality and process outcomes for both patients and ACOs; applying quasi-experimental research designs; developing novel datasets; linking existing data; and applying theory will all be important aspects of furthering the knowledge on the role of health IT within ACOs.

### Defining Relevant Outcomes

An evidentiary gap exists in studying the mechanisms through which health IT can impact health status for ACO beneficiaries or ACO performance. While the bulk of the studies we reviewed explore the outcomes of ACO participation or health IT capabilities, we found only one study focused on quality outcomes resulting from the use of health IT within ACOs [23]. More research is warranted in all of these areas, but evaluations of clinical quality and process outcomes lag behind. Researchers will need to study conditions that are “sensitive” to both health IT and the practices of accountable care to evaluate both the proposed mechanisms of ACO success and the ACO model of care. Because of the concurrent rise of health IT and delivery reform incentives [8,15], researchers should apply quasi-experimental designs and analysis methods to isolate causal effects of health IT, controlling for confounding that stems from the characteristics of early adopters [46] and ACO churn over time [1], among other factors. These designs are commonly deployed in the ACO cost-savings literature [47,48,49,50], and are relevant in the health IT arena as well.

Furthermore, while *implemented* health IT is often correlated with delivery reform participation, barriers to implementation may in turn hinder advancement of reforms like ACOs. For example, 43 percent of rural health clinics reported that lack of capital for health IT improvements impeded their ACO participation [19]. These challenges warrant studies examining specific barriers to health IT implementation or improvements among organizations that want to but may not already participate in delivery and payment reform programs. In particular, few studies have explored the implications prerequisite barriers have for the achievement of reform goals. In addition, lack of interoperability has been cited as a barrier to delivery reform efforts like ACOs [35], motivating studies to examine the effect of interoperability on ACO goals [13].

### Novel Data Needs

Much of the data necessary for the types of studies proposed above are available independently, but merging and aggregating these datasets at the ACO level is challenging. As our results show, a variety of existing data sources are ripe for studying either ACOs or health IT, but data that assesses both is limited and the unit of analysis of each varies. NSACO is perhaps the most equipped to study this intersection between health IT and ACOs. More studies using NSACO, and those using more granular information at the member organization level are needed to further this field. Part of the challenge of studying ACOs arises from their tiered nature and the complex relationships between member organizations. In order to adequately address the relationship between ACOs and health IT, it is imperative that researchers are able to link physician, patient, hospital, and other member organization information to the ACO in which they are nested. In addition, understanding the nature of the ACO contracts, proportions of shared patients, and ACO performance over time is key.

Currently, CMS provides annual performance data, ACO characteristics, and member organizations by MSSP ACO and a more specific list of skilled nursing facilities. These sources are a good starting point, but fall short of providing information on the type of organization (aside from skilled nursing facilities), number of shared beneficiaries, and organization identifiers that are linkable to other datasets such as NSACO and AHA. Finally, the use of EHD has been limited. Using EHD may add complexity to analyses, but holds the potential to more directly assess patient outcomes and clinical indicators of population health.

### Application of Theory

Most of the studies we identified did root their analyses in theoretical frameworks. Researchers use specific ACO frameworks or ground their hypotheses in organizational theories such as resource dependence theory [17]. ACOs constitute a highly complex field with different organizational structures, varied financial incentives across and within ACOs, and diverse patient populations. Due to this complexity, grounding research in theory is important both for understanding applicability to other organizations and for interpreting findings. By specifying the organizational relationships, dependencies, and constructs the investigators are measuring, organizational leaders and other researchers can better understand if the measurement of those constructs and the overall relationship definition apply to their own setting or organization. Additionally, when constructs are clearly articulated and used for hypothesis-building, researchers can more easily interpret findings within the context of the framework from which the hypotheses originated. Finally, graphical representation of theoretical models assist in model specification by illuminating potential confounding effects and the data required for reliable controls. This improves inference by isolating the causal effect of interest and identifying the data required for that isolation. While application of theory is common in the health IT and ACO literature, other streams of ACO literature like financial performance have been less inclined towards utilizing theory.

Our study has several important limitations of note. Primarily, our search strategy may have missed some articles related to ACOs and health IT. To reduce this potential issue, we took a reference snowballing approach from the papers identified in our original search. We also conducted an additional hand search to identify any papers missing from the search. We also recognize that our sample of 33 articles is fairly small for a systematic review, however given the nascence of this particular research area, we would not anticipate a large body of literature at this early stage. Finally, the studies included in our review differ in important ways, which make meta-analysis impossible and qualitative synthesis difficult. As a result, generalizability is somewhat limited.

## Conclusion

Our synthesis of the literature identified three major subgroups of health IT and ACO studies. The first studies ACO participation among hospitals and provider groups with health IT as a factor influencing participation. Second, studies looked at adoption of health IT as an outcome, with ACO participation among provider groups and hospitals as a factor in those capabilities or use of health IT. Finally, the third and smallest group represented studies examining patient or process outcomes with ACOs and health IT as factors. This orientation to the literature illustrates the need for more quasi-experimental research exploring patient and ACO process outcomes, likely using electronic health data. These data sets are not widely or easily accessible, which necessitates the need for additional research data assets. Finally, the continued use of theory in this body of literature is important for both interpretation and applicability of the findings to inform ACO decisions and advance the evidence base. Furthering the evidence-base of the effects of health IT within the context of ACOs is increasingly relevant to the ongoing discussion surrounding delivery reform.

## Additional File

The additional file for this article can be found as follows:

10.5334/egems.261.s1 Appendix. Appendix Table 1 includes the search strategy for the systematic review. Appendix Table 2 describes findings of each study by research stream identified in the review.
