# Supplementary material for: Health Information Technology and Accountable Care Organizations: A Systematic Review and Future Directions
Source: EGEMS (Wash DC). 2019 Jul 8;7(1):24. doi: 10.5334/egems.261 (PMC6625537; doi:10.5334/egems.261)
Supplement: Appendix. — Appendix Table 1 includes the search strategy for the systematic review. Appendix Table 2 describes findings of each study by research stream identified in the review. [file egems-7-1-261-s1.pdf]

Appendix

Appendix Table 1. PubMed Search Strategy

Papers meeting the following criteria were returned in a search of PubMed & MEDLINE.

**Any of the following terms related to ACOs**	**AND**	**Any of the following related to health information technology**
• Accountable care organization<br>• ACO<br>• accountable care<br>• MSSP<br>• medicare shared savings program	AND	• Health IT<br>• information technology<br>• electronic health information<br>• electronic health data<br>• electronic health record<br>• electronic medical record<br>• EHR<br>• EMR<br>• health information exchange<br>• regional health information organization

Appendix Table 2: Study Findings by Stream

Citation	Findings Relevant to ACOs & Health IT
**Stream 1: Health IT as a determinant of ACO participation or formation**	
Ortiz 2013	43% of rural health clinic leaders reported lack of available capital to invest and improve health IT systems as a barrier to joining ACOs, via survey.
Wan, 2014	Lack of capital for investment in health IT was not identified as a significant perceived barrier to participation in an ACO by managers. Similarly, having an EHR system was not a significant predictor of planned participation.
Colla, 2015	For ACOs in the NSACO survey, no association was found between those reporting a formal relationship with an outpatient pharmacy (within the ACO or via contract) and three measures of health IT. The measures were 1) the ACO reported half of its providers attesting to MU, 2) strong ability to monitor quality performance, and 3) strong ability to monitor financial performance. These three measures were grouped into "capabilities" by the authors.
Yeager, Zhang, & Diana, 2015	Hospitals who have implemented an EHR system participate in ACOs at higher rates.
Heisey-Grove & Patel, 2017	Physicians using certified EHR systems more likely to participate in ACOs (and other payment/delivery reform programs) than those who do not.<br><br>Physicians participating in a payment/delivery reform programs that use EHR for more advanced purposes are less likely to stop participating in the delivery reform program.<br><br>ACO participants are more likely to share information electronically.
Walker & Mora, 2016	Controlling for hospital characteristics, hospitals in ACOs were more likely to have HIE and electronic patient engagement. They were not more likely to attest to Meaningful Use Stages 1 or 2.
Chukmaitov, et al, 2017	Pioneer ACOs had a higher mean count of basic HIT capabilities than either MSSP or non-ACO hospitals. There was no statistically significant difference in mean basic capabilities between MSSP and non-ACO respondents. In the full model, basic HIT was negatively associated with probability of MSSP participation but advanced HIT use was positively associated with probability of MSSP participation. No significant relationship was found for advanced HIT.
Cross & Adler-Milstein, 2017	Majority of hospitals participating in an ACO also participated in some amount of information exchange with long-term care facilities. No significant relationship in full model between ACO participation and any HIE participation with long-term care organizations. ACOs were found to be more likely to participate in bidirectional-exchange with long-term care organizations than send-only exchange.
Lewis, et al, 2017 (*Soc Sci Med*)	Pre-established partnerships used to form the ACO were more likely to have all clinicians on a single EHR than those with some or all new partners. The need for

	data, analytic, or technical capabilities was identified in interviews as a reason for partnership seeking to form an ACO.
**Stream 2: Health IT as an outcome**	
Colla, et al, 2014	Physician-led ACOs more frequently reported having advanced health IT capabilities than ACOs led by non-physician organizations. There was no difference in all primary care physicians attesting to Meaningful Use by 2013 between leadership structures. Developing health IT capacity was the most commonly reported challenge to ACO implementation.
DuBois, 2014	A survey of ACOs examined readiness to optimize the use of medications to reduce costs and improve care. Results indicated wide variation regarding preparedness in medication optimization, especially in survey items exploring information technology capabilities. The optimization measures with the lowest percentage of ACOs reporting preparedness were alerting providers of potential care gaps (28%); notifying care providers when a drug is prescribed (20%); capturing patient-reported outcomes electronically (15%); and notifying care providers when an Rx is filled (9%).
Shortell, et al, 2015	Greater than 50% of ACOs use telehealth and send patient reminders electronically. 48% of ACOs provide patients with access to their medical records and an additional 24% provide patients with access to both their medical records and clinical notes.
Colla, et al, 2016	ACOs with hospitals included noted that the presence of the hospital was advantageous in achieving more advanced data sharing, especially between inpatient and outpatient settings.
King, et al, 2016	Physicians participating in an ACO or PCMH and using an EHR were more likely to participate in population management and prevention, quality measurement and reporting, care coordination, and patient engagement than those not using an EHR.
Peiris, et al, 2016	When comparing commercial and non-commercial ACOs, commercial ACOs are more likely to use a single EHR system while non-commercial are more likely to have at least 75% of primary care physicians meeting Meaningful Use criteria. No significant differences in the ability to share inpatient and outpatient data within the ACO, outside of the ACO, or having predictive risk stratification systems in place were seen between commercial and non-commercial ACOs.
Wu, et al, 2016	ACO membership in an integrated delivery system was associated with above median health IT capabilities, however, less than half of ACOs participated in a variety of practices electronically.<br>The practices with the lowest participation were integrating inpatient and outpatient data *within* the ACO (36%); predictive risk assessment and risk stratification (26%); and integrating inpatient and outpatient data *outside* of the ACO (8%).
Ali, et al, 2017	From a survey of EDs participating in ACOs in Massachusetts, 31% reported using telemedicine and 65% reported using health IT infrastructure as strategies to

	reduce spending, readmissions, or redundant testing.
Bazzoli, et al, 2017	Created taxonomies of Pioneer and MSSP ACOs. Both Pioneer and MSSP clustering resulted in 4 clusters with high basic and advanced health IT capabilities (>4.4/5 for basic, >1.7 for advanced) while one cluster for each was defined by low basic and advanced capabilities (<2.5/5 for basic, <1 for advanced).
Heisey-Grove & King, 2017 (*HSR*)	Analyzed Meaningful Use reporting data for Medicare and Medicaid providers, looking at whether or not membership in a Pioneer ACO was related to measures of Meaningful Use. Providers reporting participation in a Pioneer ACO were 1) more likely to register for MU, 2) less likely to report being paid specifically to implement health IT. Among Medicaid providers, Pioneer ACO participants were more likely to successfully attest to MU. In a separate analysis, Medicare providers participating in Pioneer ACOs were also more likely to successfully attest to MU.
Lewis, et al, 2017 (*MCRR*)	In qualitative interviews, ACO leaders identified using EHR as a strategy to improve care transformation. In particular they cited the strategies of building care processes in the EHR, including guidelines in EHRs, employing electronic alerts, monitoring protocols and guidelines, and identifying patient populations as those aided by health IT.
Markovitz, et al, 2017	Practices exposed to Meaningful Use financial incentives were more likely to participate in ACOs.<br><br>Among physician practices participating in an ACO, past experience with financial incentives or public reporting were more likely to be prepared to utilize cost and quality data.
Pimperl, 2017	Physician practice participation in an ACO associated with higher integration of Performance Management Systems as composite index of using the EHR for collecting quality measures, potential drug interactions, prompts and reminders, and alerts for abnormal test results.
Wilks, et al, 2017	Provide frequencies for ACOs scoring above 7/10 on the Medication Practices inventory. From interviews, those with greater health IT capacity cited it as a facilitator to successful medication practices while those with less health IT use cited it as a barrier.
**Stream 3: ACO Performance as a Function of Health IT**	
Larson, et al, 2012	Reviewed early progress of four Brookings-Dartmouth ACOs. Despite all having EHR systems in place to some extent, none reported interoperability across care settings. ACO leaders described health IT and data capabilities as being crucial for ACO performance.
Albright, et al, 2016	Although on average EHR capabilities did not differ between high and low-performing MSSP ACOs, greater EHR capabilities were associated with significantly better disease prevention performance.

D'Aunno, et al, 2016	ACOs identified as high performers acknowledged their EHR system and participation in a regional health information exchange as important for their performance
Schoenhaus, 2016	In an accountable care setting, after implementing a new comprehensive and EHR-based medication refill system and workflow, physicians estimated that the technology saved them between 20-30 minutes of work per day, which the authors estimate to be worth $33 to $50 of savings per day.
Stock, 2016	Within a Coordinated Care Organization in Oregon (billed as an ACO model), physicians requested in interviews that they needed more support in learning information technology and data systems used in system transformation. This study was a qualitative case study.
Wu, 2016	From interviews with leaders in ACOs, health IT is seen as fundamentally linked to many care management activities and accessible data is necessary for both clinical and financial success.
Bagwell, et al, 2017	Interviewed ACO leaders that work with rural providers. Health IT identified as important for population health management, identifying gaps in care, and reporting to Medicare. Additionally, leaders described the need to improve their IT capacity to improve performance with specific focus on interoperability between organizations and analytics.
Chukmaitov, et al, 2017	Pioneer ACOs had a higher mean count of basic HIT capabilities than either MSSP or non-ACO hospitals. There was no statistically significant difference in mean basic capabilities between MSSP and non-ACO respondents. In the full model, basic HIT was negatively associated with probability of MSSP participation but advanced HIT use was positively associated with probability of MSSP participation. No significant relationship was found for advanced HIT.
Heisey-Grove & Patel, 2017 (*JAMIA*)	Physicians who are participating in an ACO are more likely to use health IT for quality improvement, patient engagement, and population management activities. Physicians in ACOs are also more likely to share information electronically.
Huber, Shortell, & Rodriguez, 2017	ACO participation and EHR capabilities are both associated with greater care transition management capabilities.<br><br>EHR capability does not mediate the relationship between ACO participation and care transition management processes.
Kim, et al, 2017	In a qualitative single site case study of a primary care ACO in Nebraska, advanced EHR use was commonly referenced as a facilitator to successful colorectal cancer screening efforts, a commonly used quality metric for ACOs.
Wilks, et al, 2017	ACOs reporting greater health IT capacity in interviews cited it as a facilitator to successful medication practices while those with less health IT use cited it as a barrier.

Wu, 2017	Information exchange was associated with the ACO having care management processes (CMP). However, neither information capture nor information provision were associated with higher levels of CMP. Health IT capabilities associated with CMP presence were predictive risk assessment and referral information to providers.
